# A Predominant Clonal Thromboembolic Meningoencephalitis Group of *Histophilus somni* Assigned by Major Outer Membrane Protein Gene Sequencing and Pulsed-Field Gel Electrophoresis

**DOI:** 10.3389/fvets.2018.00221

**Published:** 2018-09-19

**Authors:** Yuichi Ueno, Chie Teratani, Wakako Misumi, Kaori Hoshinoo, Daisuke Takamatsu, Yuichi Tagawa, Ken Katsuda

**Affiliations:** ^1^Division of Bacterial and Parasitic Diseases, National Institute of Animal Health, National Agriculture and Food Research Organization, Tsukuba, Japan; ^2^Hyogo Prefectural Asago Livestock Hygiene Service Center, Asago, Japan; ^3^Kagoshima Prefectural Kagoshima Central Livestock Hygiene Service Center, Kagoshima, Japan; ^4^United Graduate School of Veterinary Sciences, Gifu University, Gifu, Japan

**Keywords:** *Histophilus somni*, genetic population, thromboembolic meningoencephalitis, major outer membrane protein gene sequence, pulsed-field gel electrophoresis

## Abstract

*Histophilus somni*, a member of the family *Pasteurellaceae*, causes a variety of diseases, including thromboembolic meningoencephalitis (TEME) and respiratory diseases, which result in considerable economic losses to the cattle and sheep industries. In this study, 132 chronologically diverse isolates from cattle in Japan and 68 isolates from other countries comprising 49 from cattle and 19 from sheep were characterized using major outer membrane protein (MOMP) gene sequence and pulsed-field gel electrophoresis (PFGE) analyses. The *H. somni* isolates formed nine MOMP genetic clades (clade Ia, Ib, and II–VIII) and 10 PFGE clusters (HS1–HS10). Except for two (1.0%), all isolates fell into one of the nine MOMP genetic clades, while 62 (31.0%) isolates belonged to no PFGE cluster. MOMP genetic clade Ia and PFGE cluster HS1 were the major groups, and all HS1 isolates possessed the clade Ia MOMP gene. Isolates from TEME cases were significantly associated with these major groups (chi-square test, *p* < 0.0001), as 88.2% of the TEME isolates belonged to MOMP genetic clade Ia and PFGE cluster HS1, which formed the most predominant clonal group. After an inactivated vaccine using an HS1 strain with the clade Ia MOMP gene was introduced in Japan in late 1989, the number of TEME cases and isolates assigned into the clonal group decreased simultaneously. However, the proportions of clade Ia and cluster HS1 isolates from TEME cases remained high after 1990. These results suggest a close association of TEME with PFGE cluster HS1 and MOMP genetic clade Ia, and imply the presence of factors or characteristics commonly possessed by those strains that contribute to the development of TEME.

## Introduction

*Histophilus somni* causes a variety of diseases, collectively called histophilosis, which include thromboembolic meningoencephalitis (TEME), respiratory diseases, such as pneumonia, myocarditis, and reproductive disorders, resulting in significant economic losses to the cattle and sheep industries (1–3). This organism is globally distributed and has been isolated from not only diseased tissues, but also the nasopharyngeal tracts and reproductive organs of healthy animals (1, 3). In Japan, the first clinical case of TEME caused by *H. somni* was identified in 1978 (4). Thereafter, histophilosis has been recognized as an important disease with a significant economic impact on cattle farms, and then an inactivated vaccine to prevent TEME was approved and has been used since late 1989.

*H. somni* isolates from different origins have diverse phenotypes and genotypes. Biotyping (5–7), antigenic typing (8–11), cellular protein profiling by gel electrophoresis (6), and *in vivo* virulence studies (12, 13) have been used to identify differences among *H*. *somni* strains. Riboyping (5), PCR-ribotyping (14, 15), restriction endonuclease analysis (5, 6), and genomic fingerprinting by PCR (14–16) have also been applied to assess differences among strains and to identify correlations of specific strains with specific disease phenotypes, suggesting that genetic typing would be an appropriate technique to estimate the virulence of *H. somni*. Pulsed-field gel electrophoresis (PFGE) is a standard method for the assessment of the genetic relatedness of bacterial strains (17) and several studies of *H. somni* isolates were conducted (18–20). However, these studies have failed to clarify any association between PFGE patterns and the virulence of *H. somni* isolates, probably due to the limitations of isolate numbers, isolation areas, and/or isolation sources.

The major outer membrane protein (MOMP), the most abundant surface protein of *H. somni*, has several features of the porin proteins of Gram-negative bacteria, such as the presence of 16 transmembrane β-strands and eight extracellular loops (L1-L8) of variable sequences (21). In other bacterial species, the MOMP and porin gene sequences have great diversity, which has been useful for genetic typing of bacterial isolates of *Pasteurella multocida, Haemophilus influenzae, Neisseria* spp., (22–24), and others. As with MOMP and porins of other bacterial species, MOMP of *H. somni* also has structural and sequence heterogeneity among isolates (11, 21). In fact, Tagawa et al. (11) classified 52 *H. somni* isolates from diseased and healthy animals into six groups (groups 1, 2, 3a, 3b, 3c, and 4) on the basis of the combination of molecular mass of MOMP and its antigen type as determined by the reaction patterns of five monoclonal antibodies. All isolates from diseased animals were classified into three specific groups (1, 3b, and 3c). Among them, group 1 contained all (6/6) of the TEME isolates and 80% (4/5) of the pneumonia isolates, whereas all (2/2) pleuritis and 71% (5/7) of the myocarditis isolates were classified into group 3b, and 80% (4/5) of the abortion isolates were classified as group 3c, indicating some associations between MOMP types and various diseases (11). Ward et al. (6) also suggested an association between the MOMP type and disease, as MOMP was 41 kDa in all four isolates from diseased cattle, but 33 kDa in two isolates from asymptomatic carriers.

In this study, to further elucidate the genetic diversity of *H. somni* isolates and the association of a specific genetic group with a disease phenotype, 200 *H. somni* isolates from geographically and chronologically diverse origins were analyzed using two approaches: MOMP gene sequencing and PFGE. As mentioned above, *H. somni* MOMP has antigenic and size variations (6, 11) that are ascribed to the nucleotide sequence diversity of the MOMP genes (21). Therefore, isolates with similar MOMP gene sequences may form disease-associated clades, as described in a previous study of MOMP grouping (11). PFGE could be useful to identify some associations between the genetic background and disease phenotype in large numbers of various isolates. We also compared the proportions of Japanese *H. somni* isolates with different genetic groups before and after approval of the TEME vaccine in Japan and discussed the influence of the vaccine on the current population structure of *H. somni*.

## Materials and methods

### Bacterial isolates and culture conditions

A total of 200 *H. somni* isolates from cattle (*n* = 181) and sheep (19) were characterized in this study (Table 1 and Supplementary Table S1). The cattle isolates were originated from Japan (132), the USA (21), UK (18), Switzerland (6) and Australia (4) and the sheep isolates were from the USA (2), Canada (3), and UK (14) (Table 1 and Supplementary Table S1). The type strain of *H. somni* [strain 8025(T)] was included. All of the Japanese isolates were isolated from cattle and submitted from the prefectural livestock hygiene service centers for diagnostic examination or scientific research. Four Australian isolates were isolated from cattle imported from Australia with active TEME or pneumonia during the import quarantine period. The other isolates from outside of Japan were kindly provided by Dr. L. B. Corbeil, University of California, San Diego; Dr. L. Corboz, University of Zurich; and Dr. R. Parton, University of Glasgow.

**Table 1:** Country, host, isolation year, and status of animals sampled for isolates used in this study.

			**Status of animals sampled**^**a**^											
**Country**	**Host**	**Isolation year**	**TEME**	**Res**	**Myo**	**Abo**	**Mas**	**End**	**Pru**	**Sep**	**Orc**	**Car**	**Unknown**	**Total**
Japan	Cattle	1978-1989	32	8								18	4	62
		1990-2015	13	40	6	1	3					1	4	68
		Unknown	1	1										2
USA	Cattle		4	5	1	4						6	1	21
	Sheep									1	1			2
Canada	Sheep									1			2	3
UK	Cattle			8									10	18
	Sheep												14	14
Switzerland	Cattle			1		1		1	1			2		6
Australia	Cattle		1	3										4
Total			51	66	7	6	3	1	1	2	1	27	35	200

a *Res, respiratory diseases mostly pneumonia; Myo, myocarditis; Abo, abortion; Mas, mastitis; End, endometritis; Pru, purulent semen; Sep, septicemia; Orc, orchitis; Car, asymptomatic carrier*.

All isolates were cultured on brain heart infusion agar (Difco; BD, Franklin Lakes, NJ, USA) plates supplemented with 5% defibrinated sheep blood and 0.5% yeast extract (Difco) at 37°C under 5% CO_2_ for 16 h for PFGE analysis and 20 h for PCR template preparation. All isolates were confirmed as *H. somni* by biochemical testing with established methods (25) or with a commercial biochemical identification kit (ID-test-HN-20 Rapid; Nissui Pharmaceutical Co. Ltd., Tokyo, Japan) in accordance with the manufacturer's instructions. *H. somni*-specific PCR analysis (26) was also conducted for identification of the isolates. All isolates were frozen in brain heart infusion broth (Difco) with 20% glycerol at −80°C until use.

### MOMP gene amplification and sequencing

DNA manipulations were performed as described previously (27). For DNA preparation, a few bacterial colonies were transferred from an agar plate to 500 μL of distilled water and boiled for 10 min. After centrifugation (12,000 × *g* for 2 min), 1 μL of the supernatant was used as a DNA template for PCR amplification. PCR was performed on a GeneAmp PCR System 9700 (Applied Biosystems, Foster City, CA, USA) using KOD-plus polymerase (Toyobo, Osaka, Japan) with primers (F6: 5′-AGTTCAAAAATTATTCAAAAAGTGTGATTTAGATC-3′ and R6: 5′-AGCGAAATTTTTGGCTAGCCTACC-3′) following the manufacturer's instructions. The reaction consisted of an initial denaturation step at 94°C for 2 min, followed by 30 cycles of denaturation at 94°C for 15 s, annealing at 54°C for 30 s, and extension at 68°C for 90 s, then a final extension at 68°C for 10 min. The PCR amplification products were purified using the QIAquick PCR Purification Kit (QIAGEN, Hilden, Germany) following the manufacturer's instruction and used as templates for sequencing. DNA sequencing was contracted to Hokkaido System Science Co., Ltd. or performed in the reporting facility using a DNA sequencer (3130xl Genetic Analyzer; Applied Biosystems). Six primers (F2: 5′-AAGCGGAGCATGAAGTACAA-3′, F3-1: 5′-TGGCGTTTAGCAACGCAAGT-3′, R2-2: 5′-ATCTTTTTTATGTGCTTCACC-3′, R3: 5′-CATAGTCAGCACCGACGATA-3′, and the PCR primers) were used for sequencing of the MOMP gene. DNA sequences were assembled using Sequencher ver. 4.8 Sequence Analysis Software (Gene Codes, Ann Arbor, MI, USA). Determined MOMP gene sequences were deposited in the DNA Data Bank of Japan under the accession numbers LC371064–LC371241.

The whole MOMP gene sequences of 178 isolates determined in this study and those of 22 isolates reported previously (21, 28) were translated to amino acid sequences, then aligned using the CLUSTALW algorithm (http://www.clustal.org/omega/). After retranslation of the aligned amino acid sequences into the DNA sequences, phylogenetic analysis and round-robin analysis to estimate the evolutionary divergence among the sequences were performed using MEGA 7 software (https://www.megasoftware.net/) (29). MOMP genetic clades were defined based on the topology of a phylogenetic tree as well as the evolutionary divergence as determined by the p-distance method (30). The accession numbers of the MOMP gene sequences used for analysis are described in Supplementary Table S1.

### PFGE

A few bacterial colonies were suspended in sterile NT buffer (10 mM Tris-HCl and 1 M NaCl), the suspensions were boiled for 30 s to inactivate endonucleases derived from bacterial cells. After centrifugation, the supernatant was removed and the cells were suspended at appropriate dilutions in NT buffer. The cell suspensions were added to twice the volume of 1% low melt agarose (preparative grade; Bio-Rad Laboratories, Inc., Hercules, CA, USA) in EC buffer (6 mM Tris-HCl, 1 mM NaCl, 100 mM ethylenediaminetetraacetic acid [EDTA], 0.5% Brij-58, 0.22% deoxycholate, and 0.5% sarkosyl), mixed, and solidified in disposable plug mold (Bio-Rad Laboratories). The plugs were then incubated at 37°C for 5 h in EC lysis solution (0.1 mg/mL lysostaphine [Sigma-Aldrich, St. Louis, MO, USA] in EC buffer). This solution was replaced with ESP buffer (0.5% sarkosyl, 0.5 M EDTA, and 0.5 mg/mL proteinase K [Sigma-Aldrich]) for protein digestion and incubated overnight at 50°C. The plugs were rinsed twice with TE buffer (10 mM Tris-HCl and 0.1 mM EDTA), transferred into TE buffer with 1 mM phenylmethylsulfonyl fluoride, and incubated for 1 h at room temperature. Afterward, the plugs were rinsed three times with TE buffer and stored at 4°C in TE buffer until restriction enzymatic treatment. The plugs were transferred into 200 μL of 1 × NE buffer 2.0 (New England Biolabs, Ipswich, MA, USA) with 1 mg/mL of bovine serum albumin (Takara Bio Inc., Otsu, Japan) and 20 U of *Sac*II (New England Biolabs), and incubated for 7 h at 37°C. The plugs were transferred into the wells of a 1% Pulse Field Certified Agarose (Bio-Rad) gel and electrophoresed for 20 h (2 s initial switch time to 4 s end switch time for 9 h and 7 s initial switch time to 12 s end switch time for 11 h) at 6.0 V/cm in 0.5 × Tris-borate-EDTA buffer (Sigma-Aldrich) using a CHEF-DR II PFGE system (Bio-Rad Laboratories). Lambda Ladder PFG Markers (New England Biolabs) were added to both end wells of every gel. Following separation, the gels were stained with 0.5 μg/mL of ethidium bromide solution, washed with distilled water, and photographed under ultraviolet light. The images were acquired using Quantity One 1-D Analysis Software, version 4.4.1 (Bio-Rad Laboratories).

### Cluster analysis of PFGE

Image normalization and construction of similarity matrices were carried out using BioNumerics software, version 5.1 (Applied Maths, Inc., Austin, TX, USA). DNA bands were assigned manually and a dendrogram was generated using the unweighted pair group method with an arithmetic mean (UPGMA) based on the Dice similarity index. A PFGE pattern that differed by at least one clear band was considered a distinct pattern. PFGE clusters were defined when four or more isolates share more than 80% similarity in PFGE patterns.

### Statistical analysis

Significant associations between MOMP genetic clades (clade Ia or others) and status of animals sampled (TEME or others, including carriers) or between PFGE clusters (HS1 or others) and status of animals sampled among the 162 bovine isolates with recorded clinical-pathological observations were identified using the chi-square test.

## Results and discussion

### Phylogenetic analysis of *H. somni* MOMP gene sequences and associations with status of animals sampled

**Figure 1:**
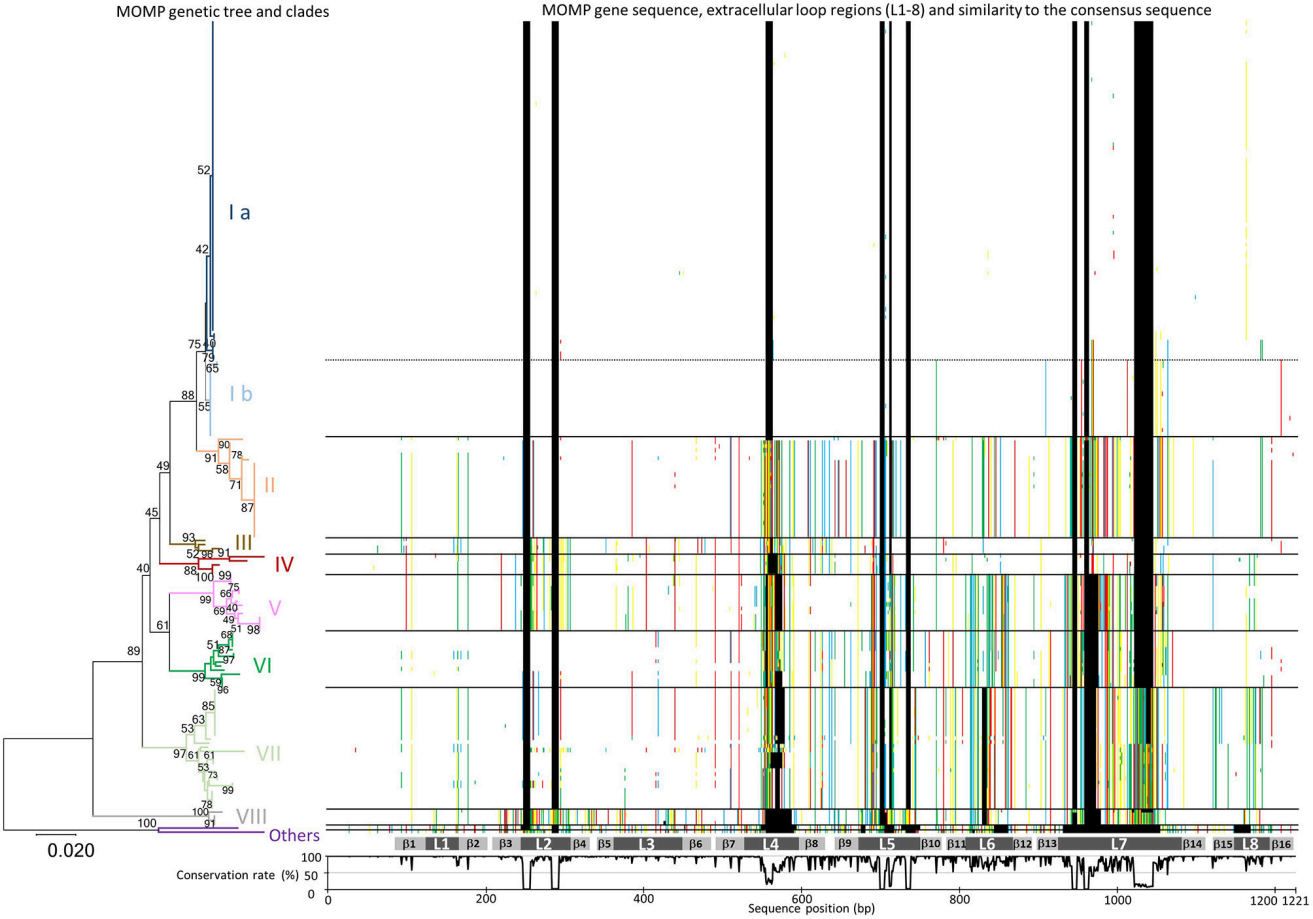
Phylogenic analysis of MOMP gene nucleotide sequences. A MOMP genetic tree was generated using the neighbor-joining method. The bootstrap values (1,000 replicates) are shown next to the branches when 40% or more. The branches of each MOMP genetic clade are colored separately. The scale bar indicates the evolutionary distance value in units of the number of base differences per site. All positions containing gaps and missing data were eliminated. Evolutionary analyses were conducted using MEGA7: Molecular Evolutionary Genetics Analysis software, ver. 7.0 (29). Sequences of MOMP genes different from the consensus sequence are colored (adenine, green; guanine, yellow; thymine, red; cytosine, blue; gaps, black) and shown next to the tree. The gray bars below the MOMP gene sequences show putative regions of the eight extracellular loops (L1–L8) and 16 transmembrane β sheets (β1–β 16). The graph under the gray bars shows the conservation rate of each consensus sequence residue.

As shown in Figure 1 and Supplementary Table S2, phylogenetic analysis of the MOMP genes revealed a high degree of sequence variation. The length of the MOMP genes ranged from 951 to 1,158 bp, and 93 different MOMP gene alleles were found among the 200 *H. somni* isolates. Insertions and deletions of three to 123 base pairs were found in seven of the eight predicted surface-exposed loop regions (L2–L8), as shown by the sequence gaps in Figure 1. Many single base substitutions were also found in the loop regions and transmembrane regions between L4 and L8, while the surface-exposed loops L1 and L3 were well conserved (Figure 1).

On the basis of the topology of a phylogenetic tree as well as evolutionary divergence, eight MOMP genetic clades (I–VIII) were defined (Figure 1 and Supplementary Table S2). The MOMP genes of two isolates (129Pt and 2-PW-L) did not belong to any clade, while all other alleles fell into one of the eight clades. The MOMP genes of bovine isolates formed clades I, II, III, VI, VII, and VIII (Figure 1 and Supplementary Table S2), while clades IV and V were constructed exclusively by the genes of 19 ovine isolates.

Among the genetic clades defined in this study, clade I was the most predominant, consisting of 102 (56.4%) of 181 bovine MOMP genes. Within the 102 clade I isolates, 17 Japanese isolates from respiratory disease, TEME, or myocarditis cases obtained in 1993 or later and two USA isolates from pneumonia and myocarditis cases appeared to form a possible subclade (clade Ib) in clade I (Figure 1), whereas the remaining 83 clade I isolates formed another subclade (clade Ia). Differences in the nucleotide sequences of the MOMP genes between these subgroups primarily occurred in the L7 hypervariable loop, suggesting that the accumulation of point mutations is the reason for these subclades. The 19 isolates of clade Ib were also clustered separately from the clade Ia isolates by PFGE analysis as shown below. The numbers of isolates in the other MOMP genetic clades were as follows: clade II, 25; III, 4; IV, 5; V, 14; VI, 14; VII, 30; and VIII, 4. Geographically, each of the MOMP genetic clades tended to be evenly distributed among the collected strains (Supplementary Table S3).

**Figure 2:**
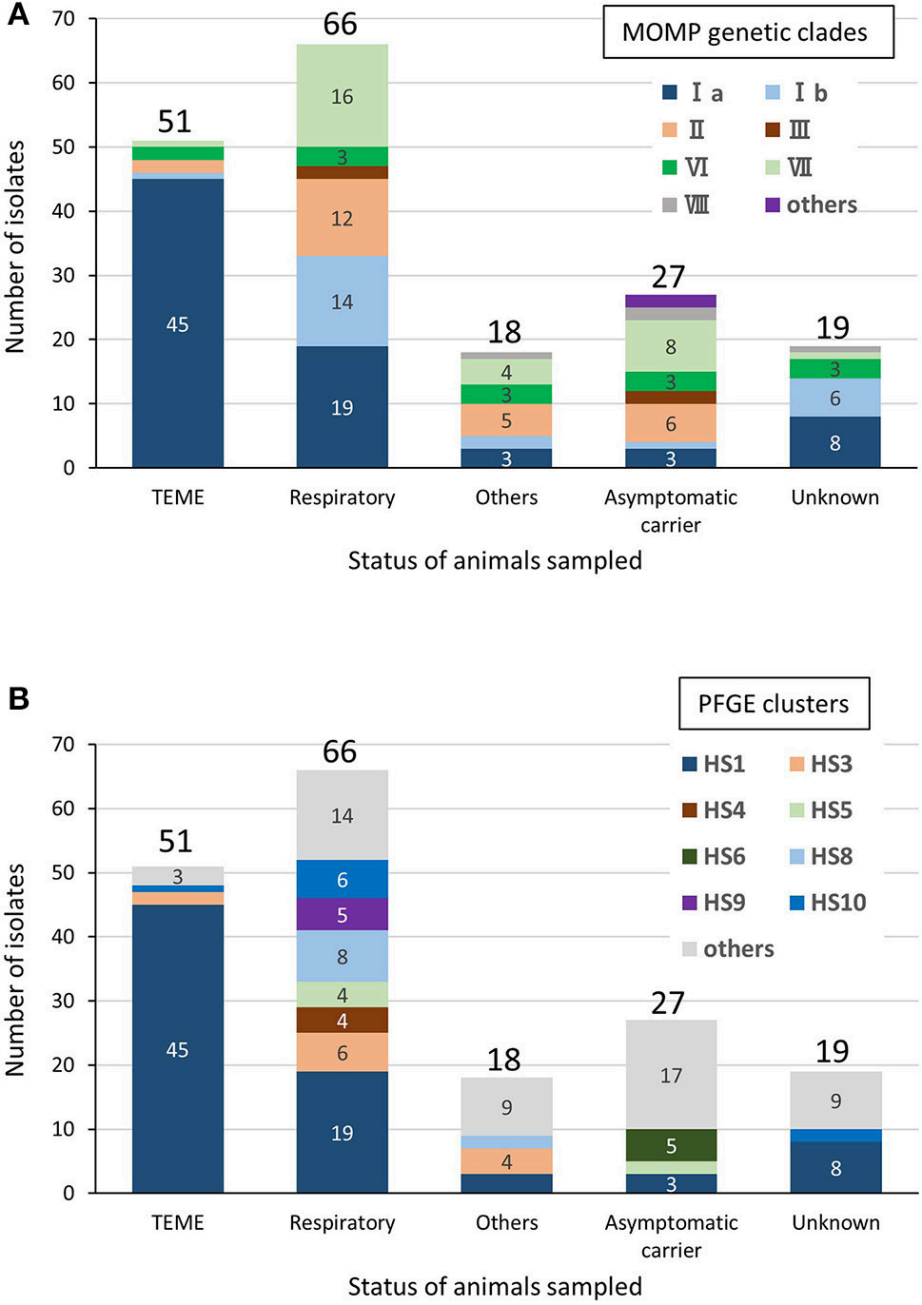
Relationships between clinical-pathological observations in cattle and MOMP genetic clades **(A)** or PFGE clusters **(B)** of isolates from cattle. “Respiratory” includes pneumonia and “Others” includes myocarditis, abortion, mastitis, endometritis, and purulent semen. The number of isolates of each status of animals sampled is shown above the bars. The number of each MOMP genetic clade with three or more isolates is shown on the bars.

Among the bovine isolates with recorded clinical-pathological observations, 45 (63.4%) of the 71 isolates in clade Ia were isolated from TEME cases, whereas the proportion of TEME isolates in the other clades was lower than that in clade Ia (clade Ib, 1/17 [5.9%]; II, 2/25 [8%]; III, 0/4 [0%]; VI, 2/11 [18.2%]; VII, 1/29 [3.4%]; and VIII, 0/3 [0%]). In addition, most of the TEME isolates (45/51, 88.2%) were assigned to clade Ia (Figure 2A), and the proportion of clade Ia isolates isolated from TEME cases was significantly higher than that isolated from non-TEME cases (25/111, 22.5%) (chi-square test, *p* < 0.0001; Supplementary Table S4). The clade Ia isolates were isolated less frequently from respiratory disease cases (19/66, 28.8%) and other diseases (3/18, 16.7%), as well as carrier animals (3/27, 11.1%; Figure 2A). These results suggest a close association between MOMP genetic clade Ia and TEME. A previous study conducted by Tagawa et al. (11) reported that 71% (5/7) of isolates from myocarditis cases and 80% (4/5) from abortion cases were classified into specific MOMP groups, respectively. However, seven isolates from myocarditis and six from abortion cases used in this study were not classified into a specific MOMP genetic clade (Supplementary Table S1). In addition, the isolates from respiratory disease cases tended to be evenly distributed among the clades (Figure 2A).

### Cluster analysis of *H. somni* PFGE patterns and associations with status of animals sampled

**Figure 3:**
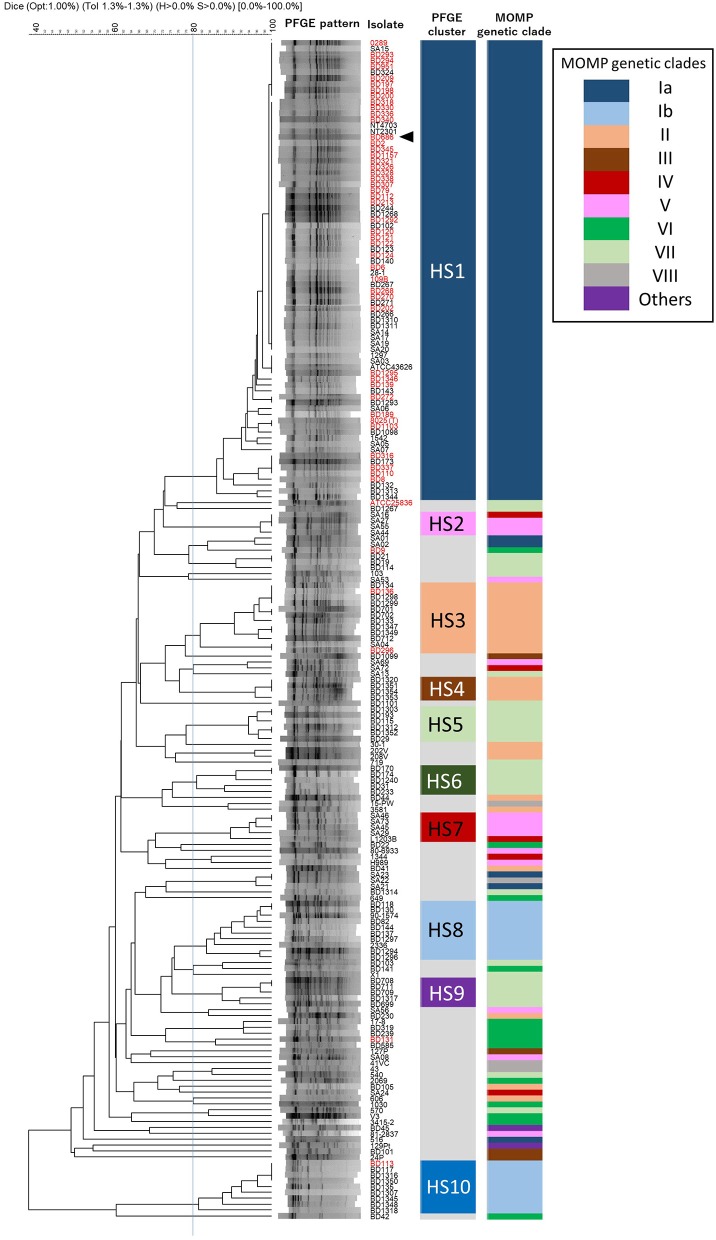
Phylogeny of PFGE patterns and the correspondence to MOMP genetic clades. A dendrogram was constructed using UPGMA clustering of Dice coefficient values. The similarity matrix was based on band-matching analysis, with optimization and tolerance settings of 1.0 and 1.3%, respectively. PFGE clusters (HS1–HS10) were defined when four or more isolates shared more than 80% similarity in the PFGE patterns. MOMP genetic clades are colored and next to the PFGE clusters. Isolates are indicated by ID numbers (Supplementary Table S1), with the exception of previously reported strains. The red-colored isolates were from TEME cases and the arrowhead indicates the vaccine strain used in Japan.

Among the 200 *H. somni* isolates, 120 different PFGE patterns were observed with *Sac*II digestion (Figure 3). To identify similarities among the PFGE patterns of *H. somni* isolates, a dendrogram was generated, and 10 distinct clusters (HS1–HS10) were designated (Figure 3). Of 200 isolates examined, 138 (69%) belonged to one of the clusters (Figure 3 and Supplementary Table S1). Cluster HS1 was the major clonal group and contained 78 (39%) isolates of diverse geographical and chronological origins. The numbers of isolates assigned to specific clusters were as follows: HS2, 4; HS3, 12; HS4, 4; HS5, 6; HS6, 5; HS7, 5; HS8, 10; HS9, 5; and HS10, 9. These small PFGE clusters consisted of isolates with some common features, as follows: UK isolates of ovine origin in HS2; pneumonic isolates from a limited area in Japan since 2009 in HS4; pneumonic or nasal isolates in Japan in HS5; nasal and vaginal isolates from carriers from a limited area in Japan in 1987 and 1988 in HS6; UK and USA isolates of ovine origin in HS7; isolates from respiratory and myocarditis cases in USA and Japan in HS8; and pneumonic isolates from a limited area in Japan in 2004 and 2005 in HS9 (Supplementary Tables S1, S5). Sixty-two (31.0%) isolates, including 39 from various countries other than Japan, were shown to be unique or formed a group of three or fewer isolates with similar PFGE patterns.

Among the bovine isolates with recorded clinical-pathological observations, 45 (64.3%) of the 70 isolates that were assigned to cluster HS1 were isolated from TEME cases. Furthermore, most of the TEME isolates (45/51, 88.2%) were assigned to cluster HS1 (Figure 2B), and the proportion of cluster HS1 isolates from TEME cases was significantly higher than isolates from non-TEME cases (25/111, 22.5%) (chi-square test, *p* < 0.0001; Supplementary Table S6), suggesting a close association of TEME with cluster HS1. As with the results of MOMP gene sequence analysis, seven isolates from myocarditis and six from abortion cases in this study were not classified into a specific PFGE cluster (Supplementary Table S1), and the isolates from respiratory disease cases tended to be evenly distributed (Figure 2B).

### Comparison of the MOMP gene sequencing and PFGE results

The correlations between MOMP genetic clades and PFGE clusters are shown in Figure 3. None of the MOMP gene sequences of the isolates in this study had a *Sac*II restriction site, indicating that the variations in the nucleotide sequences of the MOMP genes had no effect on the PFGE patterns. The PFGE patterns and MOMP genetic clades were relatively well correlated. Most of the isolates in the same PFGE cluster were classified into the same MOMP genetic clade. All isolates of the PFGE clusters HS1, HS3, HS4, HS5, HS6, HS8, HS9, and HS10 respectively possessed MOMP genes of the same genetic clade (Figure 3). But this correlation was not true in the inverse. Although most of the isolates possessing the clade Ia MOMP gene were classified into PFGE cluster HS1, isolates in the same MOMP genetic clades were frequently classified into multiple PFGE clusters. For example, isolates of MOMP genetic clade Ib were divided into PFGE clusters HS8 and HS10, isolates of clade II into HS3 and HS4, isolates of clade IV and V into HS2 and HS7, and isolates of clade VII into HS5, HS6, and HS9 (Figure 3 and Supplementary Table S1). In addition, the 200 *H. somni* isolates with 93 different MOMP gene alleles were distributed among 120 PFGE patterns. These results indicate that the discriminatory ability of PFGE is greater than that of MOMP gene sequence analysis.

Bovine isolates SA22 and SA23 had identical PFGE patterns, indicating very similar genetic backgrounds. However, the MOMP gene sequences of these two isolates were not identical: the MOMP gene of isolate SA22 was assigned to clade VIII, whereas that of isolate SA23 was assigned to clade Ia (Figure 3 and Supplementary Table S2). Three ovine isolates (SA16, SA27, and SA55) with identical PFGE patterns also possessed MOMP genes of different clades: the MOMP genes of isolates SA27 and SA55 were assigned to clade V, whereas that of isolate SA16 was assigned to clade IV. Moreover, isolates possessing identical MOMP gene sequences often had different genetic backgrounds. For example, 19 isolates of clade II had identical MOMP gene sequences (Supplementary Table S2); however, 12 and 4 of the 19 isolates were assigned to PFGE clusters HS3 and HS4, respectively, and the others were not assigned to any cluster. These results suggest the occurrence of horizontal transfer events of the *H*. *somni* MOMP genes. A previous study of *H. somni* identified possible horizontally transferable genetic regions associated with bacteriophages and transposons at several loci on the chromosomes of strains 2336 and 129Pt (31). The MOMP genes of the two strains were not located in these regions and it is unknown whether other mobile genetic elements were present near the MOMP genes. Nonetheless, since *H. somni* is known to enter a competence state for DNA transformation (32), the MOMP gene may be able to transfer horizontally among strains, even if it is not located on or near a mobile genetic element. Of note, in *H. influenzae*, which can also enter a competence state, horizontal transfer of the porin gene has been suggested (33). Together, these findings suggest that the phylogenetic relationships among *H. somni* isolates should be interpreted carefully in MOMP gene analysis.

Notably, although various PFGE patterns were observed among the ovine isolates, the MOMP genes of 19 ovine isolates were assigned to either clade IV or V (Figure 3 and Supplementary Table S1), which contained no bovine isolates. These results imply a possible role of MOMP in host specificity of *H. somni* strains, although further studies are necessary to verify this hypothesis.

### Chronological change of *H. somni* isolates support the association between TEME and isolates assigned to MOMP genetic clade ia and PFGE cluster HS1

**Figure 4:**
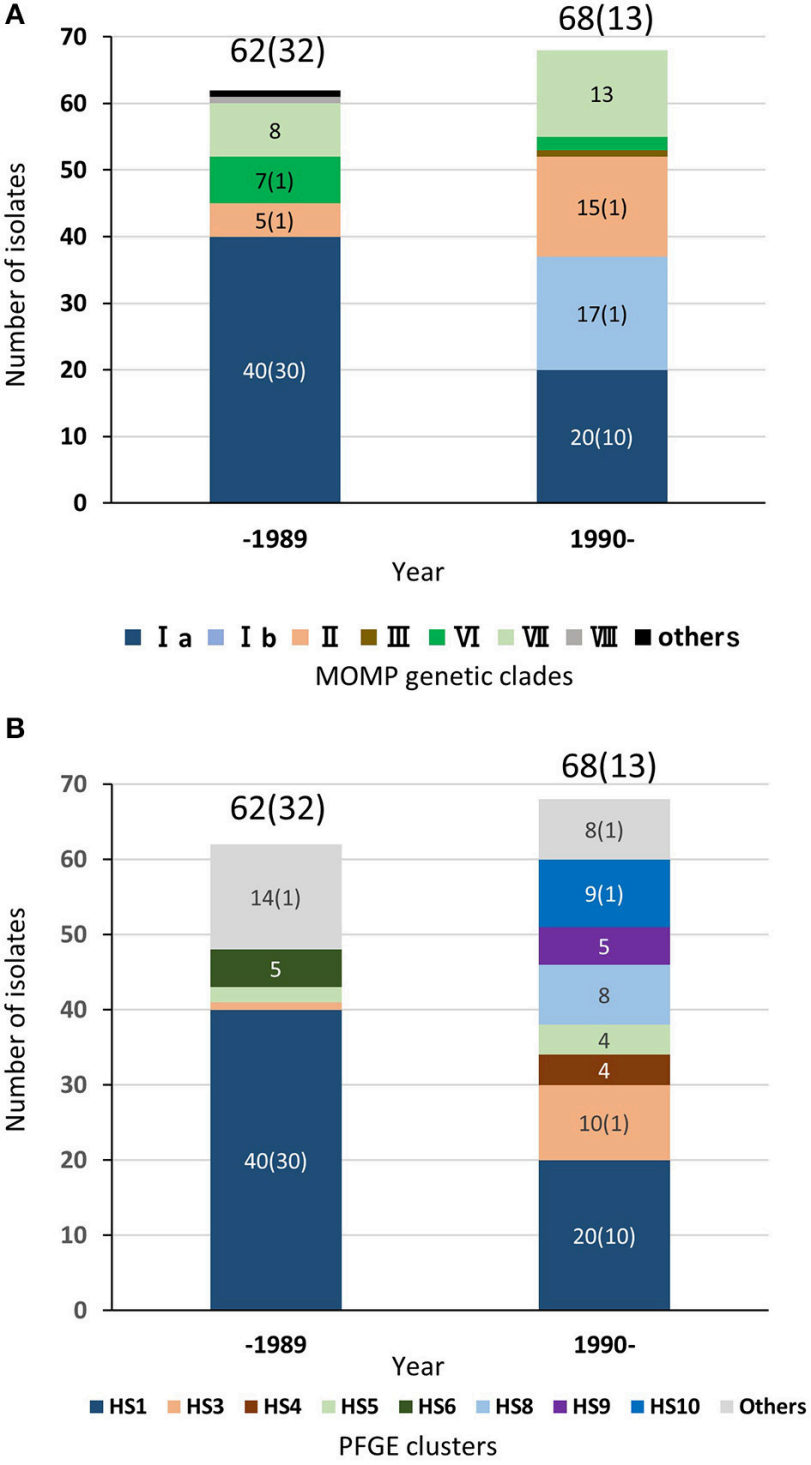
Chronological distributions of MOMP genetic clades **(A)** and PFGE clusters **(B)** of bovine isolates in Japan. In total, 122 Japanese *H. somni* isolates, of which the clinical-pathological observations and year of isolation were recorded, were used for this figure. The numbers of isolates collected prior to 1989 and since 1990 are shown above the bars. The numbers in brackets are the number of TEME isolates. The number of each MOMP genetic clade with three or more isolates is shown on the bars.

As described above, regardless of isolation year or country, most of the TEME isolates were grouped into PFGE cluster HS1, and all the HS1 isolates shared the same MOMP gene sequence as clade Ia, suggesting a strong association between TEME and these genetically related isolates. Among the 132 Japanese bovine isolates used in this study, the clinical-pathological observations and year of isolation were recorded in 122 isolates (Supplementary Table S1). In Japan, *H. somni* was first isolated from a TEME case in 1978 (4). Since then, this bacterium had been isolated mainly from TEME cases until the 1980s (Figure 4 and Supplementary Table S1). In this situation, an inactivated vaccine made from *H. somni* strain BD686 (PFGE cluster HS1 and MOMP genetic clade Ia) (arrow head in Figure 3) was approved to prevent TEME caused by *H. somni* in Japan in October 1989. Figure 4 shows the MOMP gene sequence and PFGE cluster analysis results of test isolates collected before 1989 and after 1990. The numbers of collected isolates of MOMP genetic clade Ia and PFGE cluster HS1 in our collection decreased after 1990. Also, after 1990, isolates of clade Ib and clusters HS8, HS9, and HS10 appeared in our collection at considerably high proportions. Hence, the use of the TEME vaccine may have influenced the *H. somni* population structure in Japan. However, the proportions of MOMP genetic clade Ia and PFGE cluster HS1 strains in TEME isolates remain high: the proportions were 30/32 (93.8%) before 1989 and 10/13 (76.9%) in 1990 and later. This suggests the close and constant relationship between MOMP genetic clade Ia/PFGE cluster HS1 and TEME.

For development of TEME, *H. somni* must breach epithelial barriers, reach and survive in the bloodstream, invade into the central nervous system, and cause inflammation; therefore, *H. somni* isolates of MOMP genetic clade Ia and PFGE cluster HS1 may have virulence factors contributing to these steps. Zekarias et al. (34) suggested that the retraction of alveolar epithelial cells caused by immunoglobulin binding protein A (IbpA) is a key step for the invasion of *H. somni* into the bloodstream. In fact, pneumonic lung and *H. somni* isolation from the lung were often observed in autopsy cases of clinical TEME according to the information from the prefectural livestock hygiene service centers that provided the *H. somni* isolates used in this study. Because all *H. somni* isolates from diseased cattle tested in previous studies either expressed IbpA on the bacterial surface or released it from the surface (35, 36) and the functional amino acid motif (fic motif: HPFxxGNGR) was conserved among chronologically various strains (37), most *H. somni* strains may have the intrinsic ability to damage the lung tissues and invade into the bloodstream. Corbeil et al. (38) reported that *H. somni* isolates from cattle with clinical diseases, including TEME, tended to be more resistant to bovine fresh serum than some commensal isolates from the normal prepuce. The lipooligosaccharides (LOS) of *H. somni* have also been implicated in bacterial survivability in the bloodstream (39). In addition, the LOS of *H. somni* are also expected to enhance bacterial adherence and are known to induce apoptosis of vascular endothelial cells by activating platelet cells (40, 41). Although these bacterial components may play important roles in the development of TEME, it remains to be elucidated whether there are any structural or functional variations in IbpA and LOS among strains, and whether the genetically related strains associated with TEME have particular variants of any components that are advantageous to the development of TEME.

The differences in MOMP gene sequences may affect the survivability of *H. somni* in the bloodstream of cattle. Ueno et al. (27) reported that MOMP gene exchange between *H. somni* pneumonic strain 2336 (MOMP genetic clade: Ib) and preputial strain 129Pt (MOMP genetic clade: others) affected serum susceptibility. Moreover, using mutants expressing chimeric MOMP, the authors showed that the carboxy-terminal part of the protein containing loops L4-L8 was involved in the serum susceptibility of *H. somni* (27). However, further investigations using isolates grouped into MOMP genetic clade Ia are needed to elucidate the participation of MOMP of clade Ia in the survivability of *H. somni* in the bovine blood and the development of TEME in cattle.

## Conclusions

Although *H. somni* strains show genetic diversity, our results indicates that a predominant group of strains with a specific genetic background (MOMP genetic clade Ia and PFGE cluster HS1) can cause TEME in cattle. Since the results imply the presence of virulence factors or characteristics commonly possessed by specific strains that contribute to the development of TEME, further investigations of strains of MOMP genetic clade Ia and PFGE cluster HS1 will provide novel insights into the pathogenic mechanisms of TEME associated with histophilosis.

Since both of the two molecular typing methods used in this study elucidated the genetic diversity of *H. somni* isolates tested and identified the specific group associated with TEME, both methods will be useful tools for molecular epidemiological study of this organism. Although PFGE is a time-consuming and laborious method, it has greater discriminatory ability than MOMP gene sequence analysis; therefore, PFGE will be a more powerful tool than MOMP gene sequencing for epidemiological studies of *H. somni* isolates collected in a limited period and area, such as isolates from a single outbreak. However, because of the difficulty of data sharing among laboratories, PFGE may be unfit for the large-scale analysis using previously reported data. In contrast, since it is easy to share nucleotide sequence data among laboratories using public databases, MOMP gene sequence analysis may be more appropriate as a global standard method for global-scale epidemiological studies using international *H. somni* isolates. Because each of these methods has its merits and demerits, it is important to select appropriate grouping methods according to the purpose, laboratory equipment, and time and cost available for future studies.

## Author contributions

YU, YT, and KK designed the research. YU, CT, WM, and YT performed the experiments. YU, KH, DT, KK, and YT analyzed the data. YU, KH, and YT contributed materials. All authors contributed to the preparation of the manuscript and approved the final version for publication.

### Conflict of interest statement

The authors declare that the research was conducted in the absence of any commercial or financial relationships that could be construed as a potential conflict of interest.
